# Advances in the treatment of Alzheimer’s disease

**DOI:** 10.3389/fphar.2026.1819165

**Published:** 2026-06-17

**Authors:** Rezzan Temelli Goceroglu, Ahmet Hacimuftuoglu

**Affiliations:** 1 Department of Pharmacology, Faculty of Medicine, Van Yuzuncu Yil University, Van, Türkiye; 2 Department of Medical Pharmacology, Faculty of Medicine, Atatürk University, Erzurum, Türkiye

**Keywords:** alzheimer’s disease, clinical trials, disease-modifying therapies, pathophysiology, preclinical trials, risk factors, treatment

## Abstract

Alzheimer’s Disease (AD) is a progressive neurodegenerative disease for which disease-modifying therapies remain limited. Despite extensive efforts targeting amyloid-β and tau, these approaches have not translated into clear clinical benefit, underscoring the need for a more integrated understanding of AD pathogenesis. This narrative review summarizes recent advances in the molecular mechanisms underlying AD and evaluates current and emerging therapeutic strategies. A literature search was conducted using PubMed, Google Scholar, Web of Science and Scopus, focusing on preclinical and clinical studies addressing AD pathophysiology and treatment development. Conclusion: We summarize key pathogenic pathways, including Aβ aggregation, tau hyperphosphorylation, neuroinflammation, synaptic dysfunction, and metabolic dysregulation, and discuss how these interconnected processes have informed drug development efforts. Particular attention is given to limitations of single-target approaches and the growing interest in multi-target and combination therapies. In conclusion, a better understanding of the pathological mechanisms underlying AD may contribute to the development of more effective pharmacological therapies and integrated therapeutic approaches targeting the multifactorial nature of the disease.

## 1 Introduction

### 1.1 Alzheimer’s disease

Alzheimer’s Disease (AD) is a neurodegenerative disease characterized by memory loss, progressive impairment in cognitive functions, behavioral changes, and loss of functional abilities, affecting mostly elderly people worldwide (Ana et al., 2023; Vaz and Silvestre, 2020). Clinically, the disease is characterized by a gradual decline in cognitive function, including language, memory, and simple daily activities. Among people over 65, the prevalence of AD ranges from 4% to 7%; this rate increases with age and can reach 20%–30% in the population aged 85 and over (Zhang et al., 2021; Mostafa and Asaad, 2026).

AD is the most common cause of dementia (responsible for 60%–80% of cases), and the estimated number of people with dementia is expected to reach 150 million by 2050 (Vaz and Silvestre, 2020; Abeysinghe et al., 2020). As the world population rapidly ages, AD has become a major health issue, affecting 55 million people and posing a significant emotional and economic burden on families, carers and the healthcare system (Mostafa and Asaad, 2026).

### 1.2 Method

This review was conducted following a structured literature search strategy to provide a comprehensive overview of therapeutic targets and treatment strategies in AD. Although this is not a fully systematic review, we incorporated key elements of systematic methodology and predefined search terms to improve transparency and reproducibility. A comprehensive search was performed across PubMed, Web of Science, Scopus, and Google Scholar. The search strategy included combinations of the following keywords: “Alzheimer’s disease” OR “AD”, “pathogenesis”, “pathophysiology”, “risk factors”, “biomarkers”, “amyloid-β”, “tau”, “neuroinflammation”, “neurodegeneration”, “disease-modifying therapies”, “treatment strategies”, “clinical trials”, and “preclinical studies”. Boolean operators (AND, OR) were used to refine the search.

The search was restricted to studies published within the last 5 years (2020–2025) to ensure the inclusion of the most recent evidence. However, seminal and highly cited earlier studies were also included to provide essential background context.

Studies were screened by title and abstract, with potentially relevant articles subjected to full-text review. Inclusion criteria encompassed original research articles, clinical trials, preclinical trials and high-quality review papers focusing on AD pathogenesis, biomarkers, and therapeutic strategies. We excluded non-English publications, conference abstracts without full text, and studies not directly relevant to AD. Study selection and eligibility assessment were conducted independently by the authors through consensus-based evaluation of relevance and scientific quality. Discrepancies regarding study inclusion were resolved by discussion and re-evaluation of the full text to minimize selection bias.

### 1.3 Risk factors of Alzheimer’s disease

Alzheimer’s disease (AD) is a multifactorial neurodegenerative disorder with a complex and incompletely understood pathogenesis. Late-onset AD, the most common form, typically develops in individuals >65 years (Li et al., 2018; Wang et al., 2008). Increasing age is considered the most significant risk factor for AD, particularly in the context of rising life expectancy (Mucke, 2009). In addition, several genetic, vascular, metabolic, and lifestyle-related factors have been associated with increased susceptibility to AD, including low educational attainment, traumatic brain injury, sleep disturbances, cardiovascular and cerebrovascular diseases, diabetes mellitus, obesity, hypercholesterolemia, smoking, excessive alcohol consumption, diets rich in saturated fats, depression, and hypertension (Abeysinghe et al., 2020; Bachmann et al., 2024; Anderson et al., 2024; Liu et al., 2017; Ding et al., 2024). Furthermore, dementia prevalence is higher in women and increases progressively with advancing age (Nichols et al., 2022). The apolipoprotein E4 gene allele is the most crucial genetic risk factor for late-onset Alzheimer’s disease (AD) and is associated with an increased risk of disease development (Chen et al., 2022). Most AD cases are non-familial, late-onset forms influenced by numerous genetic variants identified through genome-wide association studies (Bellenguez et al., 2020; Andrews et al., 2023). Among the currently recognized genetic contributors, the APOE ε4 variant (Bellenguez et al., 2022) has been most strongly associated with an elevated risk of developing late-onset Alzheimer’s disease. Studies indicate that APOE ε4 promotes amyloid-β (Aβ) accumulation and plaque burden in addition to new plaque formation and deposition (Chen et al., 2022; Najm et al., 2020). Furthermore, in neurons expressing APOE4, tau pathogenesis, neuroinflammation and tau-mediated neurodegeneration occur separately from Aβ pathology (Abeysinghe et al., 2020; Shi et al., 2017). Early-onset AD, a rarer form, typically occurs between the ages of 30 and 65. This is due to genetic mutations in amyloid precursor protein (APP) and Presenilin-1 (PSEN1) and Presenilin-2 (PSEN2) (Cacace et al., 2016; Lanoiselée et al., 2017) proteins (subcomponents of γ-secretase) (Abeysinghe et al., 2020).

### 1.4 Pathology of Alzheimer’s disease

**FIGURE 1:**
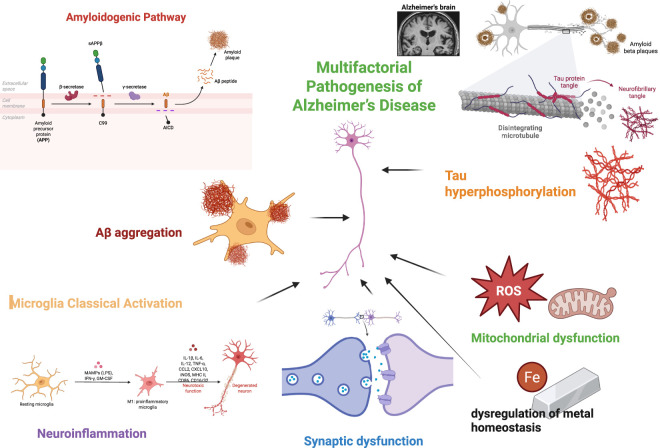
Schematic representation of the multifactorial pathogenesis of Alzheimer’s disease.

The characteristic histopathological features of AD are prominent fibrillary deposits (plaques) of amyloid beta (Aβ) peptide and neurofibrillary tangles (NFTs), the latter consisting of hyperphosphorylated tau protein (Lu et al., 2026); therefore, tau and Aβ are major drug development targets in AD (Tatulian, 2022) (Figure 1).

Amyloid precursor protein (APP) is a single-pass transmembrane protein expressed in the brain at high rates and found densely at neuronal synapses. APP, a large precursor molecule compared to Aβ, is produced by brain neurons, blood vessels, blood cells and a small number of astrocytes. APP is a protein highly expressed in the body and its levels vary with the cell’s physiological state. Although its full physiological roles remain incompletely defined, growing evidence indicates that APP contributes importantly to the regulation of neuronal survival. It plays an important role in brain development, memory and synaptic plasticity. It protects neurons and regulates intercellular connectivity, synaptic plasticity and neuronal growth. APP is transported along the axon towards the presynaptic terminal, accumulating at high rates, leading to amyloid beta (Aβ) deposition at synapses (Walsh et al., 2007; Mattson, 2004; Ma et al., 2022). Aβ, known as a neurotoxic substance, is thought to be one of the major causes of neuronal damage (Wang J. et al., 2017). It undergoes alternative proteolytic processing via two pathways: the non-amyloidogenic route involves sequential cleavage by α- and γ-secretases, while the amyloidogenic route entails β- and γ-secretase activity, leading to Aβ production. In the neurotoxic Aβ production pathway, APP is proteolytically cleaved by β- and γ-secretase, leading to the generation of Aβ peptides (Zheng and Wang, 2025). Within the amyloidogenic pathway of APP processing, sequential cleavage by β-secretase and γ-secretase, -these two membrane-associated enzymes are extracellular β-secretase (enzyme 1 (BACE1) that cleaves APP from the beta domain) and intracellular γ-secretase (a multi-domain protein with presenilin-1 (PSEN1) as its catalytic domain)-, results in the formation of Aβ peptides ranging from 37 to 43 amino acids in length, predominantly Aβ40, while the Aβ42 isoform is regarded as the most pathogenic due to its greater neurotoxic and aggregation-prone properties (Ma et al., 2022; Orobets and Karamyshev, 2023; Kurkinen et al., 2023). Mutations in the transmembrane regions of APP have been reported to alter the ratios of residues 42 and 40 of the Aβ protein, which are particularly involved in early-onset familial AD pathology (Tatulian, 2022; Ma et al., 2022). Pathological mutations in PSEN1, PSEN2 and APP are associated with early-onset AD, and account for approximately 5% of cases occurring before age 60 (Kamboh, 2018).

Aβ peptides generated through the amyloidogenic pathway induce neurotoxicity due to their ability to aggregate. Actually, Aβ monomers are prone to oligomerisation, accumulating in the form of protofibrils and fibrils, and the resulting toxicity induces oxidative stress, mitochondrial dysfunction, changes in membrane permeability, inflammation, impaired synaptic function and excitotoxicity. Additionally, Aβ disrupts cellular signalling by interfering with channels, membrane proteins and receptors (Monteiro A. R. et al., 2023; Carrillo-Mora et al., 2014).

The molecular mechanisms underlying Aβ cytotoxicity are complex and remain incompletely understood. One identified mechanism is the dysregulation of cellular ionic homeostasis, modulated by interactions with glutamatergic, cholinergic, and other ion channels in the plasma membrane of cells, or by interactions with the ryanodine receptor Ca^2+^ channel of the endoplasmic reticulum, which leads to the activation of mitochondrial permeability transition pores, causing excessive Ca^2+^ transfer to the mitochondria and apoptosis (Tatulian, 2022; Kod et al., 2018; Wang and Zheng, 2019). Direct ion-linked pores in neuronal membranes formed by Aβ have also been noted (Peters et al., 2016; Fernández-Pérez et al., 2018). Inhibition of neuronal glutamate uptake—likely mediated by Aβ dimers competing with glutamate at receptors—has been shown to cause neuronal hyperexcitability (Zott et al., 2019). Another significant mechanism is the oxidative damage in neurons that results from the production of reactive oxygen species (ROS). Aβ–metal interactions (e.g., with redox-active copper), mitochondrial Ca^2+^ overload leading to superoxide-mediated mitochondrial damage, and activation of ROS-producing enzymes such as NADPH oxidase collectively contribute to increased ROS production (Tatulian, 2022).

Tau is a microtubule-associated protein that undergoes phosphorylation and plays a crucial role in maintaining neuronal microtubule stability, cytoskeletal integrity, and axonal transport. In Alzheimer’s disease, tau pathology is characterized by abnormal hyperphosphorylation, reduced microtubule binding affinity, and aggregation into insoluble paired helical filaments (PHFs), which subsequently accumulate as neurofibrillary tangles. The formation of these pathological structures disrupts microtubule dynamics and axonal transport, impairs synaptic function, and ultimately contributes to neuronal degeneration and cell death. (Mostafa and Asaad, 2026; Tatulian, 2022; Tarafdar and Pula, 2018; Barthélemy et al., 2020).

The tau protein is a soluble microtubule-associated protein that binds to tubulin, promoting the formation and stabilization of microtubules involved in the regulation of diverse cellular functions (Peng et al., 2023). Tau promotes microtubule assembly and stability and regulates axonal migration and dendrite structure. Its biological activity is regulated by post-translational modifications, including phosphorylation at several sites (Zheng and Wang, 2025; Feng et al., 2026). In a healthy nervous system, kinase and phosphatase enzymes maintain the balance between the phosphorylation and dephosphorylation of the tau protein, thereby modulating the addition and removal of phosphate groups and their interplay with microtubules. But in a pathological state, this balance is impaired by overactivation of kinases and a decrease in phosphatase activity. These changes result in the hyperphosphorylation of the tau protein, causing tau accumulation and the production of insoluble filaments known as NFTs. This is associated with the degeneration of synapses and neurons, as well as the loss of cognitive function that is seen in patients with Alzheimer’s disease (Singh et al., 2024; Hong et al., 2025; Chen and Yu, 2023). Therefore, hyperphosphorylation of tau leads to a loss of its essential functions, and significant reductions in microtubule assembly, axonal transport, and dendritic structure, resulting in synaptic and neuronal loss and ultimately causing dementia (Monteiro A. R. et al., 2023). The progressive intracellular accumulation of hyperphosphorylated tau in selective brain regions is closely associated with Alzheimer’s disease progression and the severity of neurodegeneration. The neurotoxic pathways mediated by Aβ and tau appear to be interconnected, as Aβ monomers and oligomers have been shown to promote tau phosphorylation and subsequent intracellular tau pathology (Barthélemy et al., 2020; Mattsson et al., 2020; Amar et al., 2017; Wu et al., 2020). Microglia and astrocytes contribute significantly to Aβ and tau-mediated neurotoxicity through the promotion of neuroinflammatory responses. Extracellular accumulation of Aβ, together with intracellular tau pathology, stimulates microglial activation and the release of pro-inflammatory cytokines, including interleukins and tumor necrosis factor-α (TNF-α). These processes fuel a self-perpetuating neuroinflammatory cycle marked by astrocyte activation, increased ROS production, matrix metalloproteinase release, and further accumulation of Aβ and pathological tau. Sustained reactive microgliosis and astrogliosis ultimately contribute to synaptic dysfunction, neuronal injury, and progressive neurodegeneration (Das and Chinnathambi, 2019; Van Zeller et al., 2021).

In AD, tau is phosphorylated at more than 30 serine/threonine residues. This phosphorylation is carried out by several different kinases, such as glycogen synthase kinase-3 (GSK-3), cyclin-dependent protein kinase-5 (cdk5), protein kinase A (PKA), protein kinase C, mitogen-activated protein kinases (MAPKs) and calcium/calmodulin-dependent protein kinase II (CaMKII) (Monteiro A. R. et al., 2023; Feng et al., 2026). Aberrant regulation of serine/threonine kinases, particularly cyclin-dependent kinase 5 (CDK5) and glycogen synthase kinase-3β (GSK-3β), plays a central role in tau hyperphosphorylation in Alzheimer’s disease (Zheng and Wang, 2025; Monteiro A. R. et al., 2023; Feng et al., 2026; Maliyakkal et al., 2026). Among these kinases, GSK-3β is considered a major mediator of pathological tau phosphorylation and is closely regulated by Wnt signaling pathways (Rekha et al., 2025). In the context of AD, Wnt signaling has an important role in neurogenesis, glutamatergic synapse formation, and the proliferation, differentiation, and migration of neural precursor cells (Pang et al., 2025). Dysregulation of these signaling mechanisms contributes to microtubule destabilization, impaired axonal and peroxisomal transport, increased oxidative stress vulnerability, synaptic dysfunction, and ultimately neuronal degeneration (Feng et al., 2026; Sai et al., 2024). Therefore, like BACE-1, it is of interest as a therapeutic target for stopping or delaying the progression of the disease (Monteiro A. R. et al., 2023).

Oxidative stress is an imbalance between the production and removal of oxidants, resulting from insufficient antioxidant defenses. Consequently, excessive accumulation of reactive oxygen and nitrogen species follows. The primary consequence of this imbalance is oxidative modification of lipids (lipid peroxidation), proteins, and nucleic acids (It et al., 2019; Forman and Zhang, 2021).

Oxidative stress can cause disease progression and exacerbate symptoms through direct and indirect mechanisms. Directly, reactive species can affect cellular function, leading to cell death. Indirectly, H_2_O_2_ acting as a secondary messenger can cause disruptions in redox signaling pathways, affect normal biological functions through the modification of proteins and mitochondrial function, and facilitate inflammation, apoptosis, or autophagy. In AD, oxidative stress is associated with neurodegeneration through four main pathways: 1) Aβ production and accumulation, 2) microglial activation, 3) dysregulation of redox-active metal ions, and 4) mitochondrial dysfunction (Forman and Zhang, 2021; Circu and Aw, 2010; Savelieff et al., 2019).

Oxidative stress affects Aβ production by reducing α-secretase activity and enhancing β- and γ-secretase activities, thereby increasing senile-plaque formation Moreover, oligomeric Aβ accumulation increases oxidative stress because Aβ has been reported to induce a concentration-dependent accumulation of ROS and to enhance ROS production by directly affecting NADPH oxidase (Monteiro A. R. et al., 2023).

The biological response of the central nervous system to Aβ accumulation and tau hyperphosphorylation, through glial cell activation in the brain regions affected by AD, is another histopathological feature of the disease. Although it is known as a neuroprotective mechanism, neuroinflammation exacerbates neurotoxicity, which leads to neurodegeneration (Savelieff et al., 2019; Ganguly et al., 2017; Cai et al., 2011). Activated microglia produce both ROS and proinflammatory cytokines such as interleukin-6 (IL-6), IL-1β, and TNF-α, which initiate inflammatory responses in AD brains (Ganguly et al., 2017; Cai et al., 2011; Kwon and Koh, 2020). Inflammatory mediators released by activated microglia can also stimulate reactive astrocytes that mediate secondary inflammatory responses. Astrocytes protect and support neurons by regulating blood flow, maintaining neurotransmitter availability, and during the formation and maturation of synapses (Rinaldi et al., 2021; Juźwik et al., 2019). Nevertheless, in AD, reactive astrocytes play a key role in neuroinflammation, contributing to neuropathology and neurodegeneration, and even exacerbating it through the production of ROS and RNS. Many studies indicate the role of reactive astrocytes in the clearance of Aβ plaques. However, when the excretion capacity of the formed plaques is exceeded, or morphological changes occur, the release of Aβ from astrocytes mediates the increase in Aβ pathology. Consequently, astrocytes are emerging as a putative therapeutic target in the treatment of neurodegenerative diseases (Monteiro A. R. et al., 2023).

The concentrations of metal ions, which play important roles in various mechanisms including cell metabolism, signal transduction, protein catabolism, and stabilization, are well regulated across the blood-brain barrier (Das et al., 2021; Liu F. et al., 2022). The most redox-active metals are iron (Fe) and copper (Cu) (Ana et al., 2023; Liu F. et al., 2022). During the accumulation process, Aβ produces hydrogen peroxide, which is potentiated by Fe^2+^ and Cu^2+^ ions. Cu^2+^ ions bound to Aβ (causing it to accumulate in the form of plaques) (Xing et al., 2025) are electrochemically active and can generate ROS. These ROS cause lipid peroxidation in neuronal cell membranes, impairing glucose transport and ion channel ATPases. The oxidative stress caused by Aβ is responsible for this damage to cellular ion homeostasis and metabolism, making neurons more susceptible to apoptosis (Abeysinghe et al., 2020; Monteiro A. R. et al., 2023). Iron and copper activate GSK-3β kinase and impair synaptic function, thereby exacerbating the phosphorylation of tau protein and the formation of neurofibrillary tangles (Xing et al., 2025). Although further studies are needed to elucidate the exact mechanism of metal-Aβ neurotoxicity, there is potential for the application of iron chelation therapy in the treatment of AD (Monteiro A. R. et al., 2023; Jomova et al., 2012). Furthermore, dysregulation of metal homeostasis induces pro-inflammatory pathways in microglia, increases neuroinflammation, damages the blood-brain barrier, and disrupts synaptic function, ultimately leading to neuronal death and cognitive decline (Sharma et al., 2025). This highlights the role of metal ions in AD pathology and provides a basis for therapeutic strategies targeting metals (Xing et al., 2025).

Mitochondria play a key role in calcium homeostasis during apoptotic signaling and in cellular energy production (D'Alessandro et al., 2025). Although serving as a major intracellular source of reactive species, mitochondria are highly susceptible to oxidative stress (Ana et al., 2023). Mitochondrial dysfunction, marked by impaired oxidative phosphorylation, excessive reactive oxygen species (ROS) production, and defective mitophagy, exacerbates oxidative stress and bioenergetic failure. These pathological processes are closely interconnected, as Aβ and tau abnormalities further impair mitochondrial function, while mitochondrial dysfunction amplifies oxidative damage, neuroinflammation, and apoptotic signaling (D'Alessandro et al., 2025; Nasb et al., 2024)F. Hence, mitochondria have emerged as a potential target for disease treatment (Ana et al., 2023).

Ferroptosis is the formation of lipid peroxides and reactive oxygen species in a process dependent on iron (Qiang et al., 2024). In AD, Fe accumulation is responsible for 1) Impaired iron metabolism, glutamate excitotoxicity and the deposition of lipid-reactive oxygen species have been observed in AD tissues, which causes cellular damage (Wang et al., 2023), 2) Aβ deposition, which leads to Aβ toxicity (Yang and Nao, 2023), and 3) Tau aggregation and neurodegeneration (Spotorno et al., 2020). All these processes particularly involve Fe. Lipid peroxidation, a decrease in glutathione (GSH) levels (GSH plays essential physiological roles, including antioxidant defense and free radical scavenging, while disruption of the ROS/GSH balance can lead to oxidative damage, impaired cellular function, and cell death) (Liu T. et al., 2022), and inhibition of ferroptosis due to excessive Fe, which is detected in the brains of AD patients, have been identified as possible therapeutic strategies to delay the progression of AD (Monteiro A. R. et al., 2023).

### 1.5 Biomarkers of Alzheimer’s disease

Biomarkers are quantifiable parameters that reflect underlying biological processes or responses to therapeutic interventions (Califf, 2018) and include a range of genetic, biochemical, physiological, and neuroimaging indicators that quantify neurodegenerative processes, impaired neuroplasticity, and structural and functional brain abnormalities. These markers play a central role in advancing the understanding of AD pathophysiology, as well as in disease diagnosis, prognostic assessment, and evaluation of treatment efficacy, contributing to the identification of individuals at risk, confirmation of clinical diagnosis, prediction of disease progression and prognosis, and evaluation of treatment efficacy and safety, particularly in the context of disease-modifying therapies (DMTs). With the increasing clinical availability of DMTs, the importance of biomarkers has further expanded, as they enable the stratification of disease severity and support informed therapeutic decision-making. Consequently, the integration of biomarker-based approaches into clinical practice is essential for optimizing treatment strategies and improving patient outcomes (Jack et al., 2018; Hroudová and Fišar, 2024; Hansson et al., 2023; Pascoal et al., 2024). Biomarkers for AD encompass multiple modalities, reflecting the complexity of the disease. Among these, fluid biomarkers obtained from cerebrospinal fluid (CSF) and, more recently, blood provide valuable *in vivo* insights into underlying pathobiological processes. CSF biomarkers are widely regarded as the gold standard due to their proximity to the central nervous system; however, their clinical utility is limited by the invasive nature of CSF collection, which restricts their applicability in large-scale or routine screening settings (Bacci et al., 2026; Olsson et al., 2016). Blood-based biomarkers offer a practical and cost-effective approach, with advantages in scalability and reduced invasiveness (Schöll et al., 2024). Imaging biomarkers, including positron emission tomography (PET) and magnetic resonance imaging (MRI), provide essential *in vivo* insights into brain structure, function, and underlying pathology in AD. Among these, PET is often regarded as the reference standard against which fluid biomarkers are validated. In parallel, digital biomarkers—derived from wearable devices, smartphone-based applications, and artificial intelligence (AI)-driven platforms—are emerging as powerful tools that enable both passive and active, continuous monitoring of cognitive, motor, and functional changes in real-world settings beyond the clinical environment (Bacci et al., 2026; Erickson et al., 2025).

Cerebrospinal fluid (CSF) biomarkers for AD have been extensively investigated for over 2 decades and are widely recognized for their utility in diagnosis, prognosis, and the early detection of disease onset. Consequently, blood-based biomarkers have emerged as a promising alternative due to their minimally invasive, safe, and accessible nature. In recent years, substantial progress has been made in the development and validation of plasma biomarkers for AD, leading to the identification of several robust candidates. Notably, increased levels of phosphorylated tau (e.g., p-tau181) and decreased concentrations of amyloid-β42 (Aβ42) in blood have been associated with hallmark pathological features of AD, including neurofibrillary tangle formation and amyloid accumulation, even at early stages of the disease (Krishnamurthy et al., 2025; Rajendran and Krishnan, 2024). The three principal CSF biomarkers extensively studied in AD are amyloid-β1–42 (Aβ42), total tau (t-tau), and phosphorylated tau (p-tau). Multiple p-tau isoforms have been identified based on distinct phosphorylation sites; however, p-tau181, characterized by phosphorylation at the threonine 181 residue, remains the most widely utilized and clinically validated form (Karikari et al., 2020; Suárez-Calvet et al., 2020). The most commonly applied blood-based biomarkers in AD research and clinical settings are the Aβ42/Aβ40 ratio and phosphorylated tau variants, including p-tau181, p-tau217, and p-tau231 (Arslan et al., 2024).

The development and validation of biomarkers have been central to the approval process of new disease-targeted therapeutics (Osse et al., 2026). In the AD pipeline, disease-targeting therapies constitute the majority of investigational agents, outpacing drugs developed for symptomatic relief or neuropsychiatric conditions. This shift highlights the emphasis on disease modification and the necessity of biomarkers for both diagnostic accuracy and the demonstration of treatment efficacy (Osse et al., 2026; Cummings et al., 2025a). Among 132 disease-targeting therapy trials, 76 required a biomarker for participant eligibility, and overall, approximately 84% incorporated fluid, imaging, and/or digital biomarkers (Osse et al., 2026). The inclusion of biomarkers strengthens trial methodology by ensuring appropriate patient selection, confirming engagement of the intended molecular target, and enabling objective assessment of therapeutic outcomes. Beyond their role in clinical trials, the integration of fluid, imaging, and digital biomarkers into routine clinical practice has the potential to significantly enhance the detection, diagnosis, monitoring, and management of neurodegenerative diseases. Collectively, these advances underscore the transformative potential of biomarkers in improving clinical outcomes and advancing our understanding of disease pathophysiology (Bacci et al., 2026; Osse et al., 2026; Osse et al., 2025).

## 2 Current treatment of Alzheimer's disease

Cholinergic and glutamatergic strategies remain two therapeutic approaches in AD, but current treatments are symptomatic and do not modify the disease course (Kruk-Slomka et al., 2025). Four drugs—three acetylcholinesterase (AChE) inhibitors (donepezil, galantamine, rivastigmine) and the N-methyl-D-aspartate (NMDA) antagonist memantine—are FDA-approved (Table 1); tacrine, an earlier AChE inhibitor, was withdrawn due to hepatotoxicity (Vaz and Silvestre, 2020). Inhibition of the acetylcholinesterase enzyme increases the availability of acetylcholine and neurotransmission at synapses (Safiri et al., 2024). These agents can ameliorate cognitive and functional symptoms—donepezil, galantamine, and rivastigmine are effective in mild–moderate AD—but do not prevent progression (Alcolea-Palafox et al., 2014; Moghul and Wilkinson, 2001). Donepezil, galantamine, and rivastigmine show efficacy in mild to moderate AD. Despite minor pharmacokinetics and pharmacodynamics differences, there is no significant difference in efficacy among these drugs. These AChE inhibitors are generally well-tolerated; common side effects include nausea, vomiting, diarrhea, and anorexia (Atri, 2019; Tricco et al., 2018).

**TABLE 1:** FDA-approved pharmacotherapies for Alzheimer’s disease.

Drug	Class/Target	Mechanism of action	Indication	Key findings
Donepezil (Aricept®) FDA approved (1996)	AchE inhibitör/Cholinergic system	Enhances cholinergic neurotransmission	Mild–severe AD	Improves cognition in mild-moderate AD- Symptomatic treatment
Rivastigmin (Exelon®) FDA approved (2000)	AchE/BuChE inhibitor/Cholinergic system	Dual cholinesterase inhibition	Mild–moderate AD	Patch and oral forms
Galantamine (Razadyne®) FDA approved (2001)	AChE inhibitor + nicotinic modulator/Cholinergic system	Enhances cholinergic signaling	Mild–moderate AD	Symptomatic benefit- Dual mechanism
Memantine (Namenda®) FDA approved (2003)	NMDA antagonist/Glutamate excitotoxicity	Reduces excitotoxicity	Moderate-severe AD	Neuroprotective
Memantine + Donepezil (Namzaric®) FDA approved (2014)	NMDA antagonist + AChE inhibitor/Glutamate + cholinergic	Enhances cholinergic neurotransmission + Reduces excitotoxicity	Moderate–severe AD	Combination therapy
Aducanumab (Aduhelm®) Accelerated approval (2021)	Anti-amyloid mAb (Monoclonal antibody)/Amyloid-β	Amyloid plaque clearance	Early AD	Controversial efficacy
Lecanemab (Leqembi®) FDA approved (2023)	Anti-amyloid mAb/Soluble Aβ protofibrils	Protofibril targeting	Early AD	Slows cognitive decline
Donanemab (Kisunla®) FDA approved (2024)	Anti-amyloid mAb/Amyloid plaques	Plaque targeting	Early AD	Monthly infusion
Dextromethorphan + Bupropion (Auvelity®) FDA approved (2026)	NMDA receptor antagonist and CYP2D6 inhibitor combination	NMDA + sigma-1 modulator/Neurotransmitter modulation	AD-related agitation	Behavioral symptom treatment

AD: Alzheimer’s disease; AChE: acetylcholinesterase; BuChE: butyrylcholinesterase; NMDA: N-methyl-D-aspartate. FDA, approvals include both symptomatic therapies and disease-modifying monoclonal antibodies targeting amyloid-β pathology.

Glutamate, the predominant excitatory neurotransmitter in the brain, mediates its actions through NMDA receptors, which are crucial for learning, memory processes, and neuronal survival signaling (Wang and Reddy, 2017). Excessive activation of NMDA receptors by glutamate leads to a sustained influx of Ca^2+^ into neurons, resulting in synaptic dysfunction and ultimately neuronal death through excitotoxicity (Glaser et al., 2022), and excitotoxicity is thought to be involved in AD neurodegeneration. Memantine antagonizes NMDA receptors and reduces symptoms and neuronal damage in AD. Additionally, it regulates the Ca^2+^ imbalance induced by Aβ in cells. Therefore, this drug provides symptomatic treatment while also playing a neuroprotective role (Melnikova, 2007; DeFina et al., 2013). Memantine, when used alone, shows moderate efficacy and a safety profile in mild to severe AD. Additionally, its combination with AChE inhibitors (added to pre-existing AChE inhibitor therapy), such as donepezil, provides modest additional improvements in cognitive performance (Vaz and Silvestre, 2020; McShane et al., 2019).

**FIGURE 2:**
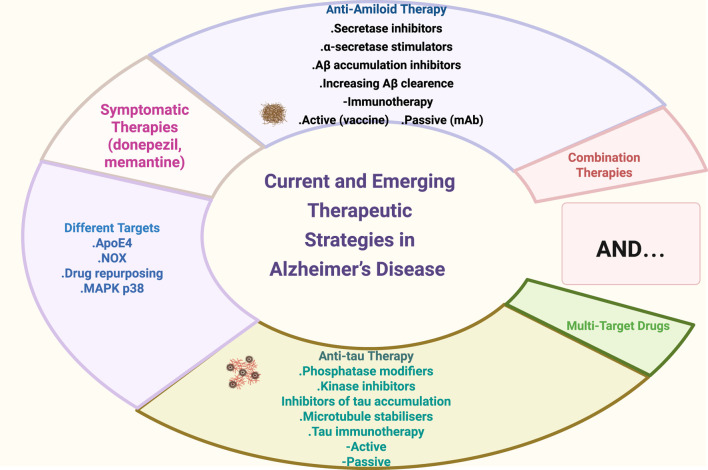
Current and emerging therapeutic strategies targeting major pathological mechanisms involved in Alzheimer’s disease.

Academic and pharmaceutical research continues to pursue the development of effective disease-modifying therapies for AD. Aβ and tau have been primary drug targets in AD for many years. The focus is now on new treatments that target different mechanisms beyond Aβ pathology. Whilst many drugs are currently being tested in clinical trials, several have been discontinued from these trials due to low efficacy or intolerable side effects. Ongoing clinical trials are focused on targets such as immunotherapy, treatments that inhibit tau protein accumulation, drugs that affect mitochondrial function, and tyrosine kinase inhibitors (Tatulian, 2022; Kruk-Slomka et al., 2025), combination therapies (Figure 2), and drug repurposing strategies.

## 3 Drug development studies in Alzheimer’s disease

Understanding the pathogenesis of Alzheimer’s Disease is a guide for developing new treatments (Yu et al., 2021). AD shows a complex multifactorial pathophysiology responsible for the disease, which is difficult to prevent. The pathogenesis of AD is associated with several complex pathways, including a deficiency in cholinergic neurotransmission, disruption in Aβ protein metabolism, accumulation and phosphorylation of tau protein (NFT), and the presence of inflammatory and oxidative pathways. Following the approval of combination therapy with donepezil and memantine for the treatment of patients with moderate to severe AD in 2014, a multi-targeted drug approach has gained the attention of researchers, with the development of hybrid molecules aimed at regulating multiple biological responses and targets. Given the complexity of AD, the combination of therapeutic agents may be more effective than monotherapy (Deardorff and Grossberg, 2016; Agatonovic-Kustrin et al., 2018; Kabir et al., 2020). Currently approved drugs are single-target. However, recent attention has shifted toward multi-treatment strategies aimed at designing and developing drugs that target multiple ways (Husain et al., 2021; Thiratmatrakul et al., 2014; Benek et al., 2020). Multi-target drugs are designed by combining the pharmacological structural features of two or more bioactive drugs into a single molecule. These pharmacophoric structural scaffolds can be combined or directly linked through a binding agent. ANAVEX 2–73 and ladostigil are examples of multi-targeted drugs. ANAVEX®2–73 (Blarcamesine) is a drug candidate that regulates cellular balance by targeting sigma-1 receptors (SIGMAR1) and muscarinic receptors. SIGMAR1 receptors are widely present in the brain (Aishwarya et al., 2021), and agonists such as blarcamesine have demonstrated efficacy in reducing neurodegenerative diseases (Jia et al., 2018). Blarcamesine, a sigma-1 receptor agonist with multi-target activity, has attracted attention due to its potential neuroprotective effects mediated through restoration of cellular homeostasis, modulation of mitochondrial function, reduction of oxidative stress, enhancement of autophagy, and attenuation of amyloid-β and tau-related pathology (Lahmy et al., 2015; Hampel et al., 2020). Furthermore, SIGMAR1 agonists regulate the effects of APP and amyloid-beta oligomers to reduce neurotoxicity (Lahmy et al., 2013). The Phase IIB/III trial indicated that blarcamesine could offer a new treatment for early-stage AD, complementing anti-beta-amyloid drugs or serving as a substitute to them (Macfarlane et al., 2025). Ladostigil, a dual inhibitor of cholinesterase and monoamine oxidase enzymes, is a hybrid conjugate of rivastigmine carbamate and N-propargyl. The second phase II clinical trials of this drug failed to prevent progression from mild cognitive impairment to Alzheimer’s disease (Athar et al., 2021). Another example of a multi-target therapeutic agent is lumateperone, which modulates several neurotransmitter systems involved in neuropsychiatric disorders. Lumateperone regulates serotonin, dopamine, and glutamate neurotransmission through multiple mechanisms, including serotonin 5-HT2A receptor antagonism, presynaptic partial agonism and postsynaptic antagonism at dopamine D2 receptors, D1 receptor-dependent modulation of glutamatergic signaling, and inhibition of serotonin reuptake (Calabrese et al., 2021; Maini et al., 2021; Titulaer et al., 2022). Lumateperone acts as a multi-target compound by modulating glutamatergic signaling in a receptor-dependent manner while simultaneously inhibiting serotonin reuptake, which may contribute to its potential neuropsychiatric benefits (Jankowska et al., 2018; Longo et al., 2023). The effect of this drug was evaluated in phase 3. However, the drug did not achieve the expected outcomes, and its phase III studies were terminated in 2018 (Snyder et al., 2015). Idalopirdine is a hybrid molecule designed to inhibit acetylcholinesterase by targeting 5-HT6 receptors. It is prepared by combining the pharmacophoric tryptamine part of 5-HT and the pharmacophoric benzylamine part of an acetylcholinesterase inhibitor. Due to its weak effect, the phase 3 clinical trials of this drug did not continue (Atri et al., 2018).

Although multi-target drugs represent a rational and promising strategy, none has yet completed clinical development and gained regulatory approval. As of January 1, 2025, a total of 182 clinical trials evaluating 138 investigational agents for Alzheimer’s disease (AD) have been identified, underscoring the continued expansion of the therapeutic development pipeline. Of these, 31 drug candidates are being assessed in 48 Phase 3 trials, 75 candidates in 86 Phase 2 trials, and 45 candidates in 48 Phase 1 trials. These studies evaluate the safety and efficacy of new molecules and repurposed drugs targeting different targets (Cummings et al., 2025a). In these clinical trials, where at least one drug targets the pathophysiology of AD, some of the targets in the pipeline for drugs in the AD drug development process are: neurotransmitter receptors (32 drugs), Aβ-related pathophysiology (25 therapeutic candidates), neuroinflammation and immune processes (24 drugs), tau-related processes (15 agents), synaptic plasticity for neuroprotection (9 drug candidates), metabolism and bioenergetics (8 agents), hormones and growth factors (5 candidates), proteinopathies, oxidative stress and vasculitis (3 candidates), gut-brain axis pathophysiology, neurogenesis and APOE and lipid and lipoprotein receptors (2 agents), circadian rhythm disorders (1 agent), 3 multi-targeted agents and 3 agents with unspecified targets. (Cummings et al., 2025a; Athar et al., 2021).

### 3.1 Anti-amyloid therapy

Amyloid plaques are formed from extracellular Aβ peptides. Aβ is derived from APP, a transmembrane protein. β secretase and γ secretase degrade APP, leading to the formation of pathological Aβ (Ono, 2018). The accumulation of Aβ results in neurotoxicity. Therefore, reducing Aβ accumulation has been a therapeutic goal for AD (Holtzman et al., 2011; Hamelin et al., 2016; Aisen, 2005). Anti-amyloid therapy consists of three strategies: secretase inhibitors, Aβ aggregation inhibitors, and Aβ immunotherapy (Yu et al., 2021).

#### 3.1.1 Secretase inhibitors

β- and γ-secretase inhibitors targeting APP cleavage have advanced to phase II and III trials, but none have achieved regulatory approval. Small molecule BACE-1 inhibitors that can pass into the brain, such as elenbecestat, atabecestat, umibecestat, verubecestat, lanabecestat, and LY3202626, have shown significant efficacy in reducing Aβ production in healthy participants and patients with prodromal, mild, and moderate AD, but phase II/III studies could not be continued due to their ineffectiveness (Ferreira et al., 2021) in slowing cognitive impairment and adverse events such as weight loss, hair discoloration, psychiatric problems and brain atrophy. (Tatulian, 2022). Verubecestat and lanabecestat were even associated with worsening of cognitive outcomes in participants with prodromal or early AD. Atabecestat, a non-selective oral BACE1 and BACE2 inhibitor, showed a dose-dependent reduction in Aβ concentration in cerebrospinal fluid but failed in phase IIb/III trials because it caused liver toxicity (due to elevation of liver enzymes) (Novak et al., 2020) and a reversible worsening of cognition in participants with mild memory loss (Wessels et al., 2020; Sperling et al., 2021). The last BACE inhibitor, elenbecestat (Das and Yan, 2019), could not continue phase II trials because it showed an unfavorable risk-benefit ratio in early AD. The phase II/III trial of Umibecestat (CNP520) was halted after participants receiving the active treatment showed a decline in cognitive function (Hampel et al., 2021). Both inhibition and modulation of γ-secretase have remained far from being therapeutic. Double-blind randomized controlled trials of γ-secretase inhibitors, including semagacestat and avagacestat, could not be continued. Drug-induced cognitive impairment has been reported (Hakem et al., 2024) in patients with mild and moderate AD with moderate cognitive impairment. The γ secretase modulator tarenflurbil also caused a worsening of cognition in moderate patients. The significant effect observed earlier was not replicated in the subsequent phase III clinical trial, with the failure of tarenflurbil largely attributed to its inadequate ability to cross the blood–brain barrier (Saretz et al., 2021). The role of secretase inhibitors is therefore a matter of debate (Yu et al., 2021). The failure of clinical trials has been attributed to incomplete understanding of BACE1 biology and physiology, limited insight into AD etiology and treatment mechanisms, and reliance on clinical endpoints without validated biomarker-based outcomes (Hampel et al., 2021). It is also noted that whilst the path towards inhibiting BACE1 to reduce pathology and improve cognitive function in AD has not proceeded smoothly, this therapeutic strategy should not be abandoned despite the difficulties encountered (Das and Yan, 2019). Presenilin, the catalytic core component of the γ-secretase complex, plays a central role in the cleavage of APP and subsequent Aβ generation (Ramachandran et al., 2021), processes strongly implicated in the pathogenesis of early-onset familial Alzheimer’s disease. Since γ-secretase also mediates the proteolytic processing of Notch receptors (Notch1, Notch2, Notch3, and Notch4), alterations in γ-secretase activity may disrupt Notch signaling pathways, potentially contributing to neurodegeneration and Alzheimer’s disease progression (Das et al., 2023). The identification of Notch as a substrate of the γ-secretase/presenilin complex, together with its recognized role in learning and memory processes, suggests a potential connection between dysregulated Notch signaling and the pathogenesis of AD (Woo et al., 2009). The Notch signaling pathway plays a fundamental role in vascular development and maintenance of vascular homeostasis. Consequently, mutations affecting Notch-related genes or disruptions in Notch signaling mechanisms have been associated with several genetic forms of vascular dementia as well as Alzheimer’s disease (Kapoor and Nation, 2021). Accumulating evidence suggests that increased Notch signaling and expression may contribute to the progression of Alzheimer’s disease, highlighting the importance of further investigating the role of this pathway in AD pathogenesis (Das et al., 2023) and supporting the potential therapeutic relevance of modulating Notch signaling through γ-secretase inhibitors (Mondal et al., 2021).

#### 3.1.2 α-secretase stimulators

Stimulation of α-secretase by protein kinase activation showed a decrease in Aβ levels (Coman and Nemeş, 2017). Etazolate (EHT 0202; ExonHit Therapeutics, Paris, France), a pyrazolopridine derivative with anxiolytic properties, is currently being investigated in clinical trials (Al et al., 2022). A Phase II trial involving patients with AD found that the agent was safe and well-tolerated (Vellas et al., 2011). Acitretin increases α-secretase secretion, and a clinical study has shown that acitretin may increase the metabolism of non-amyloidogenic APP in human patients (Endres et al., 2014). Epigallocatechin gallate (EGCG, Sunphenone) is a plant-derived polyphenolic flavonoid found in green tea. It has a broad spectrum of bioactivity, including neuroprotective properties and anti-inflammatory effects (Khalatbary and Khademi, 2020; Valverde-Salazar et al., 2023). Experimental studies have shown that EGCG reduces Aβ production (Rezai-Zadeh et al., 2005) and EGCG reduces cognitive decline by decreasing beta-amyloid levels, and that EGCG supplementation is both safe and effective for the prevention of AD in transgenic mice (Rezai-Zadeh et al., 2008). It is used as a food supplement for AD and Down syndrome. It is involved in Phase II/III studies, but the results have not yet been published (Athar et al., 2021).

#### 3.1.3 Aβ accumulation inhibitors

Small-molecule inhibitors designed to target specific Aβ oligomers (Jin et al., 2023; Pandey et al., 2024) are currently under preclinical development (Mostafa and Asaad, 2026). Despite their therapeutic potential, several limitations have hindered the successful clinical translation of these compounds. Poor penetration across the blood–brain barrier significantly restricts their bioavailability within the central nervous system, while the structural properties of many small molecules may be insufficient to effectively prevent Aβ accumulation and aggregation. In addition, protein–protein interaction surfaces often lack well-defined binding pockets, limiting the ability of small molecules to bind strongly and selectively (Yu et al., 2021; Malafaia et al., 2021). Consequently, peptide-based therapeutics have emerged as a rational and promising strategy for AD drug development, offering greater potential for selective targeting of pathological mechanisms. Reflecting this interest, multiple peptide-derived agents are currently under clinical evaluation for AD (Sehra et al., 2025).

Iron, zinc, and copper have been implicated in the regulation of BACE1 activity and the promotion of amyloid-β (Aβ) production in Alzheimer’s disease. Consequently, metal–protein attenuating compounds (MPACs) have been developed to disrupt the interaction between metal ions and Aβ, thereby potentially reducing Aβ aggregation and related neurotoxicity (Cilliers, 2021). These compounds primarily act through the chelation of metals such as copper and zinc, limiting their contribution to amyloid deposition (Sampson et al., 2014; Makin, 2018; Cheignon et al., 2018). Clioquinol, a hydroxyquinoline ionophore, is a representative example of this therapeutic strategy, as it can sequester metal ions and reduce Aβ aggregation (Alhazmi and Albratty, 2022). In individuals with mild to moderate Alzheimer’s disease, clioquinol failed to demonstrate significant benefits in cognitive measures or overall clinical outcomes when compared with placebo. Nevertheless, subgroup analyses indicated a possible slowing of cognitive decline in patients with more advanced stages of the disease. Treatment-related adverse events were reported. Although second-generation clioquinol MPAC (PBT2) appeared to offer improved safety and tolerability in moderately affected patients, it failed to produce significant cognitive or functional benefits in double-blind randomized trials of mild-moderate AD (Yu et al., 2021; Lannfelt et al., 2008).

Agents that prevent Aβ accumulation are considered a rational approach for disease modification. Targeting the early stages of Aβ aggregation, particularly the formation of soluble toxic oligomers, may represent a promising therapeutic strategy for Alzheimer’s disease by potentially preventing downstream neurodegenerative processes before extensive plaque deposition and neuronal damage occur (Sharari et al., 2023). By binding to soluble Aβ peptides, these compounds inhibit their aggregation into oligomers and thereby reduce associated neurotoxicity. Two such aggregation inhibitors that have been investigated are scyllo-inositol and tramiprosate (Vaz and Silvestre, 2020).

Scyllo-inositol can penetrate the brain and inhibit the aggregation of amyloid β-proteins into toxic fibrils and polymers (Tanaka et al., 2015). After promising results in preclinical trials (Salloway et al., 2011), scyllo-inositol has failed to show clinical efficacy in patients with mild to moderate AD in phase II trials. In addition, severe adverse effects occurred at high doses (Salloway et al., 2011; Ma et al., 2012).

Tramiprosate is an anti-amyloid agent designed to interfere with Aβ aggregation processes (Kocis et al., 2017). In preclinical Alzheimer’s disease models (Gervais et al., 2007), tramiprosate demonstrated neuroprotective potential by reducing the formation of toxic Aβ oligomers. Tramiprosate has failed to show a significant cognitive difference between treatment and control groups in phase III trials in patients with mild to moderate AD (Aisen et al., 2017; Manzano et al., 2020). Subsequent reanalysis indicated beneficial clinical effects in subgroups with ApoE4 homozygote participants (Abushakra et al., 2017). The efficacy of tramiprosate’s prodrug, ALZ-801, is currently being investigated. The phase I program showed that ALZ-801 demonstrated outstanding oral safety and tolerability in both healthy adults and elderly volunteers (Hey et al., 2018). In a 2-year phase II clinical trial involving ApoE4 carriers with early Alzheimer’s disease, ALZ-801 demonstrated favorable pharmacokinetic characteristics with no significant influence from demographic variables, supporting its potential as an orally administered disease-modifying therapeutic approach for AD (Hey et al., 2025). In a 78-week phase 3 randomized, double-blind, placebo-controlled trial involving APOE ε4/ε4 homozygous individuals (Abushakra et al., 2024), ALZ-801 demonstrated clinically meaningful cognitive benefits that were also associated with improvements in daily functioning. The findings further supported the potential role of ALZ-801 in targeting Alzheimer’s disease pathology during the presymptomatic stage in genetically high-risk populations (APOE ε4/ε4 homozygotes), emphasizing the importance of preventive therapeutic strategies. Moreover, the treatment exhibited a favorable safety profile and good long-term tolerability.

#### 3.1.4 Increasing Aβ clearance

Aβ is widely regarded as a key factor in the pathogenesis of AD, as it disrupts synaptic function and promotes neurodegeneration, thereby contributing to the cognitive decline associated with the disease (Song et al., 2022). Focusing on Aβ clearance through specific anti-Aβ antibodies represents a logical therapeutic approach. Immunotherapies directed against Aβ have emerged as some of the most promising strategies for slowing the progression of AD. At present, the most extensively developed anti-Aβ immunotherapeutic strategies include vaccines and externally administered antibodies, referred to as active and passive immunotherapy, respectively. Active immunization, which boosts or activates the host’s immune system, works by introducing Aβ or its fragments to stimulate the immune system, thereby inducing the production of endogenous antibodies targeting Aβ. Passive immunotherapy, which involves the direct administration of antibodies to the host, involves the use of humanized monoclonal antibodies or polyclonal immunoglobulins to enhance the clearance of Aβ (Song et al., 2022; Aljassabi et al., 2024). Both strategies have been explored extensively over the past decade.

##### 3.1.4.1 Active immunotherapy (vaccination)

Active immunotherapy stimulates the immune system of patients to produce antibodies against Aβ by injection of Aβ or its fragments (Vaz and Silvestre, 2020). AN-1792 was the first active Aβ vaccine to undergo clinical trials in humans; however, the trials could not be continued because meningoencephalitis developed in 6% of vaccinated patients and a strong antibody response was observed in only around 20% of them (Jin et al., 2024). The cause of the severe adverse effect observed in AN1792 is thought to be the immune response of T cells. Therefore, next-generation active immunotherapies employ modified Aβ peptides engineered to avoid T-cell activation (Yu et al., 2021). Consequently, later vaccine candidates (ACC-001, CAD106, AD02, AD03, V950, and LU AF20513) have been designed to include only unmodified or modified B-cell epitopes (Jin et al., 2024). In phase I studies, CAD106 demonstrated a good safety profile and elicited a satisfactory antibody response (Winblad et al., 2012). Phase II results further indicated a generally acceptable compromise between immunogenicity and tolerability. Nevertheless, when advanced to phase II/III as one of the earliest vaccines in this stage, CAD106 was linked to unforeseen changes in cognition, brain volume, and body weight, which resulted in premature discontinuation of the trial (Song et al., 2022). Findings from a phase II clinical trial suggested that UB-311 may have the potential to enhance cognitive performance in patients with early to mild AD (Wang C. Y. et al., 2017). A separate phase II trial with UB-311 was ultimately halted due to a mistake in treatment allocation (Song et al., 2022). On the whole, thanks to the growing body of knowledge regarding rational vaccine design and immunology, active immunotherapy targeting Aβ holds clinical prospects (Jin et al., 2024).

##### 3.1.4.2 Passive immunotherapy

Passive immunotherapy is achieved by injecting monoclonal antibodies against Aβ or its deposited forms. This approach is the most investigated strategy in clinical trials to date. (Vaz and Silvestre, 2020). Although the development of anti-Aβ immunotherapies has been marked by many failures and challenges in the pharmaceutical industry, a number of monoclonal antibody (mAb) treatments have nonetheless shown positive outcomes in phase III clinical trials and have been granted either conditional or full approval by the FDA (aducanumab, lecanemab, donanemab, crenezumab, gantenerumab, bapineuzumab, solanezumab) (Guo et al., 2024).

The initial investigation of bapineuzumab, an anti-Aβ antibody, progressed into phase III clinical trials. (Sha et al., 2026). Bapineuzumab showed promising results in early clinical studies; however, phase III trials demonstrated that bapineuzumab did not show clinical efficacy at the evaluated doses on key outcome measures in patients with mild to moderate AD (Vandenberghe et al., 2016).

Solanezumab did not meet the primary endpoints in clinical trials (Vroom and Dodart, 2024). It was found that solanezumab did not provide a significant benefit in terms of cognitive decline in patients with mild Alzheimer’s disease; furthermore, these findings failed to replicate the secondary outcomes observed in previous studies (Honig et al., 2018).

Another anti-Aβ monoclonal antibody, crenezumab, did not demonstrate a meaningful effect in slowing cognitive or functional deterioration in patients with mild to moderate AD during phase II studies. However, owing to its relatively favorable safety profile and suggested potential efficacy in earlier disease stages, a high-dose regimen of crenezumab was advanced to phase III evaluation (Cummings et al., 2018; Salloway et al., 2018). In January 2019, based on interim analyses indicating no significant improvement in cognitive or functional outcomes in phase III trials (Ostrowitzki et al., 2022). Involving prodromal and moderate AD, the studies were prematurely terminated. (Cummings et al., 2018; Roche, 2019).

Gantenerumab showed limited efficacy in slowing the progression of AD (Sha et al., 2026). Gantenerumab did not demonstrate a slowing effect on cognitive decline in symptomatic dominantly inherited Alzheimer’s disease (Salloway et al., 2021). In addition, two ongoing open-label, multicenter phase III trials of high-dose gantenerumab in prodromal and moderate AD are currently assessing the long-term safety and efficacy; the studies are expected to continue through 2026 (Sha et al., 2026; Klein et al., 2019).

In June 2021, the FDA approved the clinical use of aducanumab (BIIB037) (Aljassabi et al., 2024). Approved via the accelerated approval pathway (Llibre-Guerra et al., 2025), it represents the first new treatment authorized for AD since 2003. Aducanumab is a human IgG1 monoclonal antibody with strong affinity for Aβ, recognizing it in an extended conformation and selectively binding aggregated species such as soluble oligomers and insoluble fibrils. It reduces both soluble and insoluble forms of Aβ in a dose-dependent fashion. In individuals with mild or prodromal AD, 1 year of monthly intravenous aducanumab administration led to a dose- and time-dependent reduction in brain Aβ levels (Aljassabi et al., 2024; Sevigny et al., 2016). In August 2015, two phase III trials, ENGAGE and EMERGE, were launched to compare monthly aducanumab infusions with placebo over an 18-month period. The EMERGE study achieved statistical significance on its primary endpoint, whereas ENGAGE did not. Nonetheless, further analyses indicated that participants in the ENGAGE trial who received the high-dose regimen showed a slowing of disease progression comparable to that observed in EMERGE (Kruk-Slomka et al., 2025). However, both EMERGE and ENGAGE reported dose-dependent adverse effects (Budd et al., 2022). Nevertheless, aducanumab received FDA approval for medical use in June 2021, a decision that sparked controversy in light of the mixed and conflicting evidence on its clinical ef fectiveness (Wojtunik-Kulesza et al., 2023). Aducanumab is still being evaluated as a novel therapeutic agent in clinical trials, with limited evidence supporting its efficacy and the presence of debated adverse events such as amyloid-related imaging abnormalities (ARIA-E and ARIA-H). The U.S. Food and Drug Administration (FDA) granted accelerated approval in June 2021, with the requirement that a post-approval clinical trial be conducted to verify the expected clinical benefit of the drug. In contrast, the European Medicines Agency (EMA) withdrew its marketing authorization for aducanumab in April 2022 (Rahman et al., 2023). No clinical benefit has been demonstrated for aducanumab; the development and marketing of this drug have been halted (Høilund-Carlsen et al., 2024).

Lecanemab (BAN2401; mAb 158) is a humanized monoclonal antibody that selectively binds to soluble Aβ protofibrils (Athar et al., 2021). It is safe and well-tolerated in phase I, and preliminary results from phase II trials (NCT01767311) indicate that it significantly slows cognitive decline (Swanson et al., 2021) and reduces Aβ in the brain. Consequently, in March 2019, BAN2401 began phase III trials involving patients with prodromal and moderate Alzheimer’s Disease (Vaz and Silvestre, 2020). The phase III Clarity study of lecanemab (van Dyck et al., 2023) showed a statistically significant reduction in the rate of decline across clinical outcomes, including global, cognitive, and functional measures, in early AD, along with a marked decrease in brain amyloid levels (Vitek et al., 2023). The FDA granted traditional approval to Lecanemab in July 2023 for treating mild cognitive impairment (MCI) and mild dementia associated with Alzheimer’s disease (Beshir et al., 2024).

Other anti-Aβ antibodies in clinical trials are GSK933776, donanemab (LY3002813) and ponezumab (Vaz and Silvestre, 2020; Andreasen et al., 2015). Although ponezumab was safe and well-tolerated, its development was halted due to a lack of clinical benefit and no change in cerebrospinal fluid biomarkers (Landen et al., 2017).

Donanemab is an IgG1 monoclonal antibody that selectively targets a modified, insoluble form of Aβ that is predominantly found within amyloid plaques. In a phase III clinical trial, donanemab was shown to slow the progression of AD (Sims et al., 2023). The first regulatory approval of donanemab was granted in the United States on 2 July 2024 for patients with early symptomatic Alzheimer’s disease (Kang, 2024).

### 3.2 Anti-tau therapy

Tau protein is associated with microtubules and stabilizes microtubules in axons and dendrites, so it plays a role in controlling axonal growth and the transport processes within axons (Kimura et al., 2024). One of the two main pathological features of AD is the presence of intracellular neurofibrillary tangles (NFTs). Aberrant phosphorylation of the tau protein causes tau to detach from microtubules, leading to the formation of NFTs. Whilst there has been a greater focus in recent years on treatments targeting the production and accumulation of Aβ, findings from neuropathological and imaging studies have highlighted a stronger association between tau pathology and neurodegeneration and cognitive decline in AD, leading to an increased emphasis on treatments targeting tau (Kruk-Slomka et al., 2025; Zhang et al., 2025). Anti-tau therapies include phosphatase modifiers, kinase inhibitors, inhibitors of tau accumulation, microtubule stabilizers, and tau immunotherapy (Yu et al., 2021).

Phosphatase modifiers reduce phosphorylation by activating phosphatases such as protein phosphatase 2A (PP2A). Sodium selenate, a PP2A activator, is an important molecule in neuronal function (Soeda and Takashima, 2020; Pillai et al., 2014). Sodium selenate has been shown to reduce tau phosphorylation in animal models and in neuronal cells (Rueli et al., 2017; Corcoran et al., 2010). In a phase II trial involving sodium selenate, sodium selenate treatment was generally safe and well-tolerated but did not show any change in cognitive performance in patients with mild to moderate AD (Malpas et al., 2016). Two phase IIb trials are currently evaluating the safety, tolerability, and efficacy of sodium selenate, with completion anticipated in 2025 (Congdon et al., 2023).

The hyperphosphorylation of abnormal tau protein, driven by an imbalance between kinase and phosphatase activities (Singh et al., 2024) is regarded as a key event in tau pathology. Many protein kinases are involved in tau protein phosphorylation. Approaches aimed at reducing tau hyperphosphorylation predominantly target glycogen synthase kinase 3-beta (GSK-3β) -an enzyme that phosphorylates tau-as a key enzyme (Feng et al., 2026). Dysregulation of GSK-3β leads to hyperphosphorylation of tau and subsequently to neurodegeneration in AD. Therefore, inhibition of GSK-3β is considered a rational strategy in the treatment of AD (Vaz and Silvestre, 2020).

Tideglusib, an irreversible inhibitor of GSK-3β (Domínguez et al., 2012). In a phase IIa trial (Del Ser et al., 2013) that includes 30 patients with mild to moderate AD treated with AChE inhibitors, tideglusib (400–1,000 mg/day), showed an acceptable safety profile. While improvements in cognitive measures and clinical outcomes were observed, these effects were not statistically significant. In another phase II trial, tideglusib was generally well tolerated but did not demonstrate any clinical benefit in this trial (Lovestone et al., 2015).

Lithium, a widely used treatment for bipolar disorder, has also been investigated in Alzheimer’s disease because of its inhibitory effect on GSK-3β (Dell'Osso et al., 2016; Hampel et al., 2019). In a clinical trial investigating lithium in mild AD patients, lithium did not change cognitive performance, and the results of the study did not provide any evidence to support the view that lithium treatment might result in a decrease in the excessive phosphorylation of the tau protein (Hampel et al., 2009). In contrast, more recent phase II trials in patients with prodromal AD have demonstrated that lithium can reduce levels of hyperphosphorylated tau in cerebrospinal fluid and improve cognitive performance (Forlenza et al., 2011). A separate small-scale study using microdoses of lithium (300 μg daily) reported a deceleration in cognitive decline (Nunes et al., 2013). Additional research is required to clarify the clinical benefits of lithium salts in AD.

The spread and accumulation of tau aggregates are linked to neuronal loss and clinical impairment in AD. Accordingly, inhibiting tau aggregation has been considered a rational approach for disease-modifying treatment in AD (Vaz and Silvestre, 2020).

Methylthioninium chloride (known as methylene blue, the oxidized salt of methylthioninium) has been shown to inhibit tau aggregation *in vitro* (Isaev et al., 2024). A phase II clinical trial (Wischik et al., 2015) reported cognitive benefits in patients with mild to moderate AD; however, the high-dose regimen showed limited absorption and was poorly tolerated when administered without food. These findings led to the development of leucomethylthioninium bis (LMTM) (hydromethanesulfate), a reduced derivative of methylene blue. This molecule is more stable and has better absorption and tolerability. In a phase III trial (Gauthier et al., 2016) involving patients with mild to moderate AD, LMTM did not slow cognitive or functional decline. However, a reanalysis of the data indicated that LMTM monotherapy might still offer potential benefits. Studies are ongoing (Vaz and Silvestre, 2020).

Curcumin is a colored dietary supplement that inhibits tau aggregation by reducing the formation of the β-sheet form of tau *in vitro* and promoting the degradation of tau oligomers (Rane et al., 2017). In a Phase II study, daily oral curcumin supplementation led to improvements in memory and attention in adults without dementia; the study findings indicated that the improvement in symptoms was associated with a reduction in amyloid and tau deposits in the brain regions that regulate mood and memory (Small et al., 2018). A second phase II study (NCT01811381) is investigating the effects of curcumin, administered alone or in combination with yoga, in individuals with MCI or subjective cognitive impairment. Although the study was planned for completion in 2020, the results have not yet been published (Congdon et al., 2023).

The microtubule-associated protein tau (MAPT) gene encodes tau, a neuronal protein involved in the regulation of microtubule assembly, stabilization, and axonal transport within the central nervous system, and MAPTRx (ISIS 814907/BIIB080) is a tau-targeting antisense oligonucleotide (ASO) developed to selectively reduce the expression of MAPT messenger RNA and consequently lower tau protein production (Mummery et al., 2023). Antisense oligonucleotides (ASOs) consist of short, chemically engineered single-stranded DNA-like molecules that regulate gene expression by binding complementary RNA targets through Watson–Crick base-pair interactions (Vandermeulen et al., 2024). By selectively targeting specific mRNA sequences, ASOs can suppress the production of distinct protein isoforms, making them particularly valuable for disorders characterized by aberrant or disease-specific protein variants (Mansour and El-Khatib, 2025). Cell-based and animal studies have shown that small interfering RNA (siRNA) and ASOs can reduce tau expression (Monteiro A. R. et al., 2023). ASOs directed against MAPT mRNA to lower tau expression have shown encouraging outcomes in preclinical studies (Sud et al., 2014), including reductions in cerebrospinal fluid tau levels and decreased tau PET signal in clinical trials. Additional results from ongoing phase II (BIIB080 -phase II, NIO752-phase Ib) ASO studies are still awaited (Dahiya et al., 2025; Dudzisz and Wandzik, 2025).

#### 3.2.1 Increasing tau clearance

In recent years, tau immunotherapy has gained interest in response to the limited success of Aβ-targeted strategies. Both active and passive immunization approaches seek to generate or deliver antibodies against the tau protein. These strategies are designed to specifically recognize pathological tau conformations while sparing normal tau function. By promoting the removal of abnormal tau species, such interventions are anticipated to slow neuronal degeneration and alleviate clinical manifestations (Bittar et al., 2020; Sandusky-Beltran and Sigurdsson, 2020).

##### 3.2.1.1 Active immunotherapy

Anti-tau vaccines stimulate the patient’s immune system to generate antibodies directed against pathogenic forms of tau (Plotkin and Cashman, 2020).

AADvac1 is the first anti-tau vaccine tested in clinical trials. In a phase I trial which was the first-in-human study, AADvac1 demonstrated a favorable safety profile along with strong immunogenicity (Novak et al., 2017). In another Phase I trial, AADvac1 demonstrated a favorable safety profile. The clinical trial noted that the trend in IgG titres during periods without vaccination suggested a need for more frequent booster doses; it was also noted that, in patients with high titres, MRI assessments showed a tendency for atrophy to progress more slowly, and cognitive assessments revealed less decline, which is also promising (Novak et al., 2018). In September 2019, the first results of the phase II trial were announced. AADvac1 demonstrated positive immunogenicity (98.2% of patients developed specific anti-tau antibodies) and was confirmed to be safe and well-tolerated. In addition, AADvac1 induced statistically significant changes in biomarkers in blood and cerebrospinal fluid, indicating its effect in slowing the progression of tau pathology (Phase, 2019). These positive results support the transition to phase III and highlight the need for larger, well-stratified trials to determine the vaccine’s clinical effectiveness (Congdon et al., 2023).

ACI-35 is another anti-tau vaccine. A phase Ib study confirmed its safety; however, the induced immune response was limited (Wu et al., 2025). A second-generation vaccine, ACI-35.030 (JNJ 2056), was designed to induce antibodies that selectively target pathological phosphorylated tau in the brain. The phase Ib/IIa trial in early AD has been completed, with interim findings indicating that ACI-35.030 elicited robust antibody responses against both p-tau and paired helical filaments (PHFs) (Sol et al., 2025).

##### 3.2.1.2 Passive immunotherapy

In passive immunotherapy, monoclonal antibodies directed against abnormal tau proteins are administered. This approach is generally considered safer than tau vaccination, which has led to a greater number of studies exploring this strategy (Plotkin and Cashman, 2020).

E2814 (Etalanetug) is a humanized IgG1 antibody that shows high affinity for the microtubule-binding region of the tau protein (Wu et al., 2025). A phase I trial indicated good tolerability, favorable pharmacokinetic (PK) properties, and minimal immunogenicity, with no treatment-related serious adverse events reported (Rawal et al., 2025). It is currently under investigation in the phase II/III trial for Dominantly Inherited Alzheimer’s Disease (DIAD), as well as in a phase II study (Kimura et al., 2024) for early sporadic AD, where it is also being combined with anti-amyloid therapy (lecanemab) to evaluate safety, tolerability, and potential disease-modifying effects (Arshad and Alladi, 2025).

Gosuranemab (BIIB092) was safe and well-tolerated in phase I trial and there were no reports of severe adverse events (Qureshi et al., 2018). The phase II trial assessed the safety and efficacy of gosuranemabin patients with Alzheimer’s disease (Shulman et al., 2023). Gosuranemab showed effective target engagement however it failed to improve cognitive or functional outcomes and was linked to an accelerated decline in cognition relative to placebo (Wu et al., 2025). As a result, both the trial and the clinical development of gosuranemab were halted (Congdon et al., 2023).

JNJ-63733657 (posdinemab) is a humanized IgG1/kappa monoclonal antibody developed to selectively bind tau phosphorylated at threonine 217 in the proline-rich region (Wu et al., 2025). In the phase I study, the safety, tolerability, pharmacokinetics, and immunogenicity were evaluated in healthy volunteers and in patients with prodromal or mild AD. The antibody was found to be generally well tolerated, with a dose-dependent decrease in p-tau levels. No safety or tolerability concerns were identified (Galpern et al., 2024). A phase II study (NCT04619420) investigating its efficacy in early AD is still ongoing (Wu et al., 2025).

Tilavonemab (ABBV-8E12) is another monoclonal antibody in phase II trials. Phase II study of tilavonemab was carried out in patients with early-stage AD, but failed to slow disease progression or improve cognition in early AD patients (Florian et al., 2023). Owing to the absence of efficacy across all studies, the development program for tilavonemab was discontinued (Congdon and Sigurdsson, 2018).

Semorinemab (RO7105705) has demonstrated an acceptable safety profile in healthy individuals. Semorinemab did not reduce brain tau accumulation or slow the progression of clinical decline in a phase II AD trial (Teng et al., 2022). Another Phase II study suggests that semorinemab may have a modest beneficial cognitive effect in patients with mild to moderate AD. However, it has been noted that this potential cognitive benefit does not lead to an improvement in functional or overall outcomes and may therefore fail to meet the regulatory standards expected of a disease-modifying drug for AD (Monteiro C. et al., 2023).

In October 2021, Lilly terminated phase II trials of zagotenemab due to a lack of efficacy (Tatulian, 2022).

Other anti-tau antibodies that have reached phase I trials are RG7345 and BIIB076. BIIB076 was ultimately discontinued in July 2022 due to business considerations (Congdon et al., 2023). The studies of RG7345 were terminated by Roche (Congdon and Sigurdsson, 2018).

Passive tau immunotherapy with Bepranemab (UCB0107), has been reported to slow the progression of tau pathology and cognitive decline. Although the primary endpoints were not achieved in the overall study population, the trial demonstrated a biological reduction in tau accumulation accompanied by a significant, moderate improvement in cognition, supporting the potential benefit of tau-targeted therapies (Courade et al., 2025).

### 3.3 Agents with different targets

Apart from Aβ and tau, the APOE ε4 allele is considered an important biomarker as it is a key genetic risk factor (Bellenguez et al., 2022) for late-onset AD. Carriage of the APOE ε4 allele is linked to an estimated 6% higher risk of cognitive impairment, emphasizing its contribution to the development of cognitive decline. (Poblano et al., 2024). HAE-4–mediated passive immunotherapy, an antibody that selectively targets the non-lipidated forms of human ApoE3 and ApoE4 found in amyloid plaques and cerebral amyloid angiopathy (CAA), has been shown to reduce parenchymal Aβ pathology in mice expressing human ApoE4 (Liao et al., 2018; Gratuze et al., 2022) Furthermore, HAE-4 therapy decreased both amyloid plaque burden and CAA without inducing the microhemorrhages often observed with other anti-amyloid antibodies, and these results indicated that APOE-directed interventions at the core of CAA and plaques could reduce amyloid burden while maintaining cerebrovascular structure and function (Xiong et al., 2021). Targeting ApoE4 is emerging as a promising approach for the treatment of late-onset Alzheimer’s disease (Poblano et al., 2024).

As a glutaminyl cyclase inhibitor, Varoglutamstat inhibits the production of pyroglutamate Aβ (pEAβ), a species that is significantly increased and particularly toxic in AD brains (Xu et al., 2019). Clinical evaluation of Varoglutamstat has been completed for Phase I and early Phase IIa trials with successful outcomes. The Phase IIa study investigated varoglutamstat in patients with mild cognitive impairment (MCI) or mild dementia due to AD, focusing on its safety, tolerability, and efficacy (Feldman et al., 2024). The findings demonstrated a favorable safety and tolerability profile (Scheltens et al., 2018). At present, two Phase 2 clinical trials are underway to assess varoglutamstat in patients with AD (Feldman et al., 2024).

Aβ is known to interact with multiple distinct cell surface and intracellular receptors, leading to impaired cell signaling (Tatulian, 2022). Elayta is a novel therapeutic candidate for AD, acting as a brain-penetrant small-molecule sigma-2 receptor antagonist that prevents Aβ oligomer binding to neurons by redirecting them into the cerebrospinal fluid (CSF) (Steinfield et al., 2025). In a phase Ib/IIa trial (Izzo et al., 2021), no adverse events affecting participant safety were observed. In another phase I trial treatment was well tolerated (Grundman et al., 2019). Clinical trials in phase II are ongoing (Rasheed et al., 2022).

The mitogen-activated protein kinase p38, which is involved in the phosphorylation of tau, has neuropathogenic (p38a and p38b) or neuroprotective (p38c) roles depending on the isoform-specific site of phosphorylation (D'Mello, 2021). The mitogen-activated protein kinase (MAPK) pathway is a major regulator of glial-mediated neuroinflammation, involving both microglia and astrocytes. In particular, p38α MAPK promotes NF-κB activation, contributing to glutamate excitotoxicity and impaired synaptic plasticity. It is activated by pro-inflammatory stimuli, including cytokines, chemokines, and lipopolysaccharide (LPS), and has also been implicated in tau phosphorylation (Detka et al., 2024; Thakur et al., 2023). As a small-molecule selective inhibitor, neflamapimod targets p38α MAPK, an enzyme abundantly expressed in microglia and other brain cells (Detka et al., 2024). In preclinical studies, neflamapimod was demonstrated to modulate microglial activation, improve mitochondrial function and synaptic transmission, and consequently enhance memory. (Alam, 2015; Uddin et al., 2020). In a phase 2 trial, neflamapimod did not improve episodic memory in patients with mild AD. However, treatment with neflamapimod reduced CSF biomarkers of synaptic dysfunction. Combined with the study’s pharmacokinetic–pharmacodynamic data, these findings indicate that neflamapimod should be evaluated at a higher dose and for a longer duration to determine its effects on AD progression (Prins et al., 2021). Moreover, neflamapimod is being assessed in phase 2 studies for its efficacy in patients diagnosed with dementia with Lewy bodies (Alam et al., 2023; Jiang et al., 2022). MW150, a p38αMAPK kinase inhibitor, is currently being evaluated in phase II clinical trials as a potential treatment for AD (Paidlewar et al., 2024). Aberrant regulation of cyclin-dependent kinase 5 (CDK5) has been strongly implicated in the pathophysiology of Alzheimer’s disease, making it a promising therapeutic target (Garemilla et al., 2024). Dysregulated CDK5 activity contributes to tau hyperphosphorylation, Aβ accumulation, synaptic dysfunction, mitochondrial impairment, neuronal death, and abnormal cell cycle activation (Lazarevic et al., 2017; Sheng et al., 2016; Lari et al., 2025). In particular, CDK5/p25 dysregulation influences APP metabolism and abnormal APP phosphorylation, thereby enhancing Aβ production (Alam et al., 2023). The truncated CDK5 activator p25 has also been associated with several neurodegenerative disorders, including AD (Garemilla et al., 2024). Formation of the hyperactive and stable CDK5/p25 complex promotes abnormal phosphorylation of cytoskeletal proteins such as neurofilaments and tau, ultimately contributing to neurodegeneration and neuronal loss (Madar et al., 2024). Furthermore, studies in cellular and animal models have demonstrated that inhibition of the p25/CDK5 complex can attenuate tau-mediated pathology and neurodegeneration, highlighting the therapeutic potential of targeting p25-mediated CDK5 dysregulation in AD (Seo et al., 2017; Pao et al., 2023; Allnutt et al., 2020).

As an enzyme producing ROS, NADPH oxidase (NOX) is critically involved in oxidative stress and neuroinflammatory processes, with elevated levels observed in MCI and AD brains in association with Aβ accumulation. (Ganguly et al., 2021). Several small-molecule or protein-based drug candidates that inhibit NADPH oxidase are being investigated for mild cognitive decline and halting the progression of AD (Ganguly et al., 2021; Fragoso-Morales et al., 2021). NOX2 and NOX4 are the major NADPH oxidase isoforms associated with AD pathology, due to their elevated expression and activity in specific brain regions (Sorce et al., 2017). The contribution of NOX isoform-derived ROS to AD pathogenesis highlights the importance of understanding their underlying mechanisms, which may enable new treatment strategies for the disease (Kim and Moon, 2024).

As a widely recognized strategy in drug development, drug repurposing—also termed drug repositioning—has long been utilized in practice (Pattanaik et al., 2025). In the 2025 Alzheimer’s disease drug development pipeline, 46 repurposed agents are present, corresponding to 33% of all compounds in clinical trials. These agents constitute 39% of Phase 3 candidates, 41% of Phase 2 candidates, and 16% of Phase 1 candidates. Phase III includes 21 trials of repurposed agents, representing 44% of phase III trials, and 12 repurposed drug candidates, accounting for 39% of phase III agents (Cummings et al., 2025a). Many of these agents are approved for indications such as cancer (e.g., masitinib, lenalidomide) and cardiovascular or hypertensive diseases (e.g., amlodipine, losartan, telmisartan), while others investigated include drugs for neurological disorders (e.g., levetiracetam, mirtazapine), diabetes (e.g., metformin, benfotiamine), and additional conditions (Tatulian, 2022). There are also approved drugs that have been tested for their potential effects on AD in preclinical studies. COX-1 and COX-2 enzymes play a role in promoting neuroinflammation and synaptic loss within the central nervous system (Joodi et al., 2025). Treatment with celecoxib, as a selective COX-2 inhibitor, was shown to reduce memory impairment and increase neurogenesis in rats injected with aluminium chloride (Aal et al., 2021). The main receptors for cysteinyl leukotrienes (CysLTs) are CysLT1R and CysLT2R. LTD-4 binding to CysLT1R triggers β-secretase activation and promotes neuroinflammatory processes in the hippocampus and cerebral cortex (Lai et al., 2014). In scopolamine-injected rats, montelukast, a CysLT1R antagonist used in asthma treatment, alleviated memory decline and supported neuronal growth (Yerraguravagari et al., 2024); it is also undergoing Phase II clinical evaluation (Pattanaik et al., 2025). Treatment with lapatinib ditosylate, an epidermal growth factor receptor–targeting anticancer drug, reduced Aβ and tau levels in D-galactose–induced cognitive impairment in rats, while also suppressing NADPH oxidase–mediated ROS production and p38 MAPK activity (Mansour et al., 2021; Mansour et al., 2022). Several growth factor inhibitor compounds are being trialed as potential treatments for AD (Mansour et al., 2022). As a calcium channel blocker, diltiazem lowers acetylcholinesterase activity and oxidative stress, leading to improved outcomes in AlCl_3_-induced dementia in mice (Rani et al., 2015), and further mitigates intra-cerebroventricular-streptozotocin (ICV-STZ)-induced cognitive impairment (Alluri et al., 2023). In the ICV-STZ mouse model, Verapamil mitigated astrogliosis and synaptic dysfunction while improving cognitive performance (Ahmed et al., 2021). Phosphodiesterase-5 (PDE-5) inhibitors, including Sildenafil, originally used for erectile dysfunction, improved memory deficits in APP/PS1 transgenic mice (Zhang et al., 2013), while tadalafil reversed cognitive abnormalities and promoted neuronal growth in STZ-induced models (Salem et al., 2021). In line with this, preclinical studies have also been conducted with antidiabetic drugs -it has been reported to stimulate neuronal growth and improve synaptic transmission, while also decreasing Aβ42 accumulation, neuroinflammation, and apoptosis (El-Sahar et al., 2021; Yossef et al., 2020; Pilipenko et al., 2020)-metformin, an antidiabetic agent, is currently being evaluated in phase III clinical trials (Luchsinger et al., 2025; Cummings J. et al., 2024)-, antidepressants -they have been suggested to alleviate cognitive impairment and promote neuronal growth- (Krishna et al., 2021; Zhou et al., 2019)-escitalopram is being evaluated in phase III clinical studies (Rajji et al., 2025)- and antiepileptic drugs-it has been reported that these agents enhance recognition and spatial memory through the reduction of β-secretase activity, Aβ42 accumulation, tau hyperphosphorylation, and microglial activation, alongside increased α-secretase cleavage- (Alavi et al., 2022; Sorial and El, 2017). Levetiracetam, an atypical antiepileptic drug, improved cognitive decline in streptozotocin (STZ)-injected rats. It inhibited tau aggregation, acetylcholinesterase (AChE) activation, neuroinflammation, and oxidative damage induced by STZ (Alavi et al., 2022). Furthermore, levetiracetam is currently in phase III clinical trials (Cummings J. et al., 2024).

Microtubules participate in a range of cellular functions, including the intracellular transport of proteins, organelles, synaptic vesicles, and other macromolecules. Under normal conditions, tau acts as a microtubule-stabilizing protein; however, its hyperphosphorylation results in the loss of this stabilizing function. The resulting disruption of microtubule integrity and axonal transport is considered to contribute significantly to neurodegeneration in AD (Vaz and Silvestre, 2020).

Davunetide (NAP) is a peptide that stabilizes microtubules in neurons and glial cells (Ivashko-Pachima et al., 2017), although its precise mechanism of action remains unclear (Gozes et al., 2018). Encouraged by favorable preclinical findings, davunetide was advanced into clinical trials (Varidaki et al., 2018). In a phase II trial involving intranasal administration in prodromal AD patients, the treatment was well tolerated; however, no statistically significant improvement in cognitive performance was observed (Morimoto et al., 2013).

Epothilone D (EpoD) is an antifungal agent with microtubule-stabilizing properties (Robles-Gómez et al., 2023). It facilitates the polymerization of tubulin into microtubules and increases microtubule organization *in vitro*. Although it improved working and spatial memory deficits in aged tau transgenic mice, a phase I clinical trial evaluating safety and pharmacodynamic outcomes was discontinued because of intolerable adverse effects. After its termination in 2013, no further clinical development was pursued (Vaz and Silvestre, 2020; Yu et al., 2021).

TPI-287 (abeotaxane) is a microtubule stabilizer that has demonstrated safety and efficacy in clinical trials for cancer (Congdon and Sigurdsson, 2018). In a double-blind, randomized clinical trial involving patients with mild to moderate AD, the treatment group showed a modest reduction in the decline of Mini-Mental State Examination scores compared with placebo. However, serious adverse events, including anaphylactic reactions, were reported (Tsai et al., 2020), and no clinical trials of this drug are currently ongoing (Congdon and Sigurdsson, 2018).

Due to the complex and multifactorial pathology of AD, interventions aimed at multiple biomarkers may offer greater therapeutic efficacy, either through drug combinations or the development of multi-target agents. Combination therapies that offer a strategic approach to concurrently target multiple pathological pathways involved in disease progression (Cummings et al., 2025a; Cummings J. L. et al., 2024; Fillit et al., 2025), are classified as pharmacodynamic or pharmacokinetic based on their mechanisms. Pharmacodynamic combinations involve two or more treatments with additive or synergistic effects. These may include multiple drugs, drug combinations with other interventions (e.g., devices or lifestyle changes), natural therapies, or single agents that act on multiple targets or pathways (Angioni et al., 2025). Pharmacokinetic combination approaches comprise the co-administration of one or more active agents with adjunctive compounds that modulate their absorption, distribution, metabolism, or excretion (ADME), thereby enhancing systemic exposure, minimizing peripheral toxicity, and improving central nervous system (CNS) delivery via blood–brain barrier (BBB) modulation (Cummings J. L. et al., 2024). Twenty clinical trials conducted in 2025 involved combination drug therapies (Cummings et al., 2025b). Targets for combination therapies are not limited to amyloid-related mechanisms but also include tau dysregulation, inflammatory pathways, neurodegenerative cascades, and overlapping proteinopathies such as alpha-synuclein and transactive response DNA-binding protein-43 (TDP-43) (Cummings et al., 2025b). Several ongoing clinical trials (Table 2) are now evaluating combination-based therapeutic strategies in Alzheimer’s disease, particularly approaches combining anti-amyloid therapies with anti-tau agents, metabolic modulators, or senolytic compounds (Cummings et al., 2025a; Fillit et al., 2025). These studies reflect a growing shift from single-target interventions toward multi-pathway modulation in AD. Overall, ongoing clinical trials highlight the potential of combination therapies to provide additive or synergistic effects by addressing multiple disease mechanisms simultaneously, thereby offering a more comprehensive therapeutic approach to Alzheimer’s disease (Angioni et al., 2025). With the advent of new treatment options, combination therapy is poised to play a central role in mitigating the diverse clinical features of AD, encompassing cognitive, behavioral, and functional outcomes (Cummings J. L. et al., 2024).

**TABLE 2:** Several ongoing combination therapy trials in Alzheimer’s disease.

NCT No	Combination/Intervention	Main target	Phase/Design	Population-status	Results
NCT06602258	E2814 + lecanemab	Anti-tau + anti-Aβ	Phase II	Early AD- Active, not recruiting	No results have been submitted or published to date. Primary Completion Date (Estimated): Dec 3, 2026
NCT05269394	E2814 + lecanemab	Anti-tau + anti-Aβ	Phase II/III-DIAN-TU	Dominantly inherited AD- Active, not recruiting	No results have been submitted or published to date. Primary Completion Date (Estimated): Apr, 2028
NCT01760005	Lecanemab + E2814	Anti-Aβ + anti-tau	Phase II/III-DIAN-TU	Dominantly inherited AD- Active, not recruiting	Primary Completion Date (Estimated): Apr, 2028
NCT06996730	Donanemab + RG6289	Aβ clearance + production	Phase II/III-Combination study	Autosomal-dominant AD- Not yet recruiting	No results have been submitted or published to date. Primary Completion Date (Estimated): Jun 1, 2030
NCT06957418	Regimen A: AADvac1Regimen B (Tau2) ± donanemab	Tau + Aβ	Phase II- Platform trial	Early AD-Not yet recruiting	No results have been submitted or published to date. Primary Completion Date (Estimated): Aug 31, 2028
NCT06072963	Intranasal insulin + semaglutide	Metabolic	Phase II	MCI/high risk-Recruiting	No results have been submitted or published to date. Primary Completion Date (Estimated): Dec 1, 2027
NCT04063124	Dasatinib + quercetin	Senolytic	Phase I/II-Pilot	Early AD- Completed in	Gonzales et al. (2023)
NCT04044131	Metabolic combination (NAC + others)	Mitochondrial	Phase II-Clinical trial	AD-Completed in	Yulug et al. (2023)
NCT04570644	ALZT-OP1(ALZT-OP1 (cromolyn and ibuprofen) ALZT-OP1a (cromolyn) and ALZT-OP1b (ibuprofen))	Neuroinflammation	Phase I/II	AD-Completed in	No results have been submitted or published to date
NCT05109169	MET-FINGER: Metformin + FINGER 2.0 multidomain lifestyle-based intervention	Cognition	Phase IIb Multi-national Randomised, Controlled Trial	At risk of dementia- Recruiting	No results have been submitted or published to date. Primary Completion Date (Estimated): 2Feb 28, 2027
NCT01872598	Masitinib + cholinesterase inhibitor (donepezil, rivastigmine or galantamine) and/or memantine vs. placebo	Neuroinflammation	Phase III	Mild to moderate AD-Completed in Dec, 2020	No results have been submitted or published to date
NCT05511363	KarXT (xanomeline + trospium chloride)	Psychosis associated with Alzheimer’s Disease	Phase III	AD-Recruiting	No results have been submitted or published to date. Primary Completion Date (Estimated): Oct 5, 2026

A total of 127 investigational agents are currently being evaluated in 164 clinical trials for (AD). The FDA approved new drugs, including lecanemab and donanemab, in 2023–2024. Although these drugs show promise in clearing amyloid plaques from the brain, the clinical benefits of these antibodies remain limited; they can only slow or delay the disease progression and do not treat or cure the disease. Therefore, there is a significant need for a better understanding of the mechanisms that trigger and cause AD (Zheng and Wang, 2025; Xing et al., 2025). Because of the multifactorial and complex nature of Alzheimer’s disease, therapeutic success remains limited, with failure rates reported to be as high as 95%. Indeed, approximately 99% of drug candidates have failed to demonstrate clinical efficacy, leading to the discontinuation of their development programs. These repeated setbacks have resulted in substantial disappointment across the field, prompting some pharmaceutical companies, such as Pfizer, to withdraw from Alzheimer’s disease research altogether. Meanwhile, others, including Eli Lilly, Merck & Co., and Johnson & Johnson, have discontinued specific therapeutic approaches, particularly those targeting β-secretase (BACE) inhibition (Tatulian, 2022).

## 4 Limitations

This review has several limitations. Alzheimer’s disease is a highly complex and multifactorial disorder, which inherently complicates the interpretation of current therapeutic and biomarker evidence. In addition, the existing literature remains predominantly focused on amyloid-related mechanisms, potentially underrepresenting other important pathological pathways, including tau pathology, neuroinflammation, and metabolic dysfunction. The persistent discrepancy between preclinical success and clinical efficacy further highlights the challenges of translating experimental findings into effective therapeutic interventions. Moreover, the high failure rate observed in AD clinical trials continues to complicate the interpretation of therapeutic outcomes. The rapidly evolving nature of AD research, particularly with the emergence of disease-modifying therapies and advanced biomarker technologies, may also influence the long-term applicability of some findings presented in this review.

From a methodological perspective, the literature search was limited to PubMed, Google Scholar, Web of Science, and Scopus, which may have resulted in the exclusion of relevant studies indexed in other databases. Study screening and selection were conducted through consensus-based evaluation of relevance and scientific quality; however, some degree of selection bias and subjectivity cannot be entirely excluded. Finally, the level and maturity of evidence vary substantially across different areas of AD research. While established aspects of AD pathology are supported by robust clinical and experimental data, emerging therapeutic and biomarker approaches remain less developed and therefore could only be discussed in broader terms, which may affect the overall consistency of the review.

## 5 Conclusion

In our review, we aimed to discuss the pathological mechanisms involved in AD and the therapies and therapeutic approaches targeting these mechanisms from a pharmacological perspective. Alzheimer’s disease is driven by multiple interacting pathological processes, and an incomplete understanding of how these pathways converge remains a major barrier to effective therapy development. Although amyloid-β and tau have driven much therapeutic research, clinical outcomes have often been limited, highlighting the need to target additional contributors such as neuroinflammation, oxidative stress, mitochondrial dysfunction, and synaptic failure. Future treatment strategies will likely require earlier intervention, biomarker-guided patient stratification, and combination or multi-target approaches to slow progression and preserve cognitive function. Well-designed, adequately powered trials with clinically meaningful endpoints will be essential to translate mechanistic advances into durable benefits for patients.
